# Biosurfactant production by a *Bacillus megaterium* strain

**DOI:** 10.1515/biol-2020-0068

**Published:** 2020-09-01

**Authors:** Mihaela Marilena Stancu

**Affiliations:** Institute of Biology Bucharest of Romanian Academy, 296 Splaiul Independentei, Bucharest, 060031, P.O. Box 56-53, Romania

**Keywords:** *Bacillus megaterium*, biosurfactant production, *n*-hexadecane, nitrogen, carbon sources

## Abstract

The aim of the present study was to investigate the ability of *Bacillus megaterium* IBB_Po17_ (GenBank KX499518) cells to produce biosurfactant when the growth was done in the presence of long-chain *n*-alkane *n*-hexadecane on medium supplemented with yeast extract, proteose peptone, starch, or cellulose. *B. megaterium* IBB_Po17_ revealed a higher growth in the presence of *n*-hexadecane when the medium was supplemented with yeast extract, proteose peptone, or starch, compared with cellulose. Biosurfactant production was higher when *B. megaterium* IBB_Po17_ was grown in the presence of *n*-hexadecane on yeast extract, proteose peptone, or starch supplemented medium, compared with biosurfactant produced on cellulose supplemented medium. A direct correlation between cell growth and biosurfactant production was observed. When the growth of *B. megaterium* IBB_Po17_ cells was higher, the decrease in pH values of the medium was higher too, and more amount of CO_2_ was released. Changes in cell morphology, aggregation of the cells in clusters, and biofilm formation were observed when *B. megaterium* IBB_Po17_ was grown in the presence of *n*-hexadecane on medium supplemented with yeast extract, proteose peptone, starch, or cellulose. Due to its physiological abilities, this Gram-positive bacterium could be a promising candidate for the bioremediation of petroleum hydrocarbon polluted environments.

## 1 Introduction

*Bacillus megaterium* is a genus of Gram-positive, spore-forming, motile, mainly aerobic chemoheterotrophic bacterium belonging to the family Bacillaceae. Like other *Bacillus* strains, *B. megaterium* shows a wide range of physiological abilities, such as endospore formation and production of essential antibiotics, which allow the organism to grow and survive in widely diverse habitats (e.g., soil, seawater, sediment, petrochemical wastes) [1,2]. This nonpathogenic bacterium can grow in simple media on various carbon sources. Its ability to grow on various inexpensive carbon sources makes this bacterium an ideal industrial protein production host. Furthermore, *B. megaterium* strains, which usually carry multiple plasmids, are excellent hosts for gene expression [1]. The most important products occurring in *B. megaterium* are proteins like penicillin acylase, amylases, neutral protease, dehydrogenases, P-450 cytochrome monooxygenases, and vitamins with many biotechnological and industrial applications [1,2,3]. P-450 cytochrome monooxygenases are hemoproteins involved in a variety of reactions, including in the conversion of alkanes, terpenes, or aromatic compounds, and carbon source assimilation [3]. Several *Bacillus* species produce biosurfactants (also known as surface-active compounds), which are used in numerous industrial and environmental fields, including in petroleum industries and bioremediation of petroleum hydrocarbon polluted environments [4,5]. In the last few decades, there has been an increased scientific interest in the isolation of bacteria, which produce tensioactive molecules with excellent surfactant characteristics, low toxicity, and high emulsifying activity [6,7]. Biosurfactants are produced mainly by aerobic bacteria when they are grown in liquid media with several carbon sources. It is believed that biosurfactants are secreted into the culture medium to assist the growth of different bacterial strains by facilitating the transport and translocation of insoluble hydrophobic substrates (e.g., hydrocarbons, oils) across the cell membranes [8]. Most of the biosurfactants are produced from water-insoluble substrates, although many of them are obtained through soluble substrates (e.g., carbohydrates) or a combination of both [6,8]. Therefore, *B. megaterium* is an interesting microorganism due to its broad distribution in environments, biochemical versatility, protein secretion system, and its effectiveness as an industrial protein production strain [1].

The objective of the present study was to investigate the ability of *B. megaterium* strain IBB_Po17_ (GenBank KX499518) to produce biosurfactant when the growth was done in the presence of different nitrogen (i.e., yeast extract, proteose peptone) or carbon (i.e., starch, cellulose) sources. Long-chain *n*-alkane, such as *n*-hexadecane, with a high logarithm of the partition coefficient of the hydrocarbon in the octanol–water mixture (log *P*_OW_ value 9.15), was used as the hydrophobic carbon source in this study.

## 2 Materials and methods

### 2.1 Bacterial strain and culture conditions

*Bacillus megaterium* strain IBB_Po17_ (GenBank KX499518) was inoculated into a nutrient-rich LB (Luria–Bertani) medium. The flask was incubated for 24 h at 30°C on a rotary shaker (200 rpm). Bacterial cells were harvested by centrifugation, washed twice, and finally the cell pellets were resuspended (OD_660_ 0.07) in basal medium (pH 7.2) containing: 0.1% K_2_HPO_4_, 0.1% KH_2_PO_4_, 1% NaCl, and 0.25% MgSO_4_, and supplemented with 0.5% nitrogen (i.e., yeast extract, proteose peptone) or 0.5% carbon (i.e., starch, cellulose) sources. Long-chain *n*-alkane, such as 5% (v/v) *n*-hexadecane, was finally added to the cell suspensions. The flasks were sealed and incubated for 72 h at 30°C on a rotary shaker (200 rpm).

Reagents used in this study were purchased from Merck (Darmstadt, Germany), Sigma-Aldrich (Saint-Quentin-Fallavier, France), or Bio-Rad Laboratories (Hercules, CA, USA).

### 2.2 Growth experiments

The cell growth was monitored by determining the optical density at 660 nm (OD_660_) using a SPECORD 200 UV-visible spectrophotometer (Analytik Jena, Jena, Germany). Briefly, the bacterial cells were harvested by centrifugation, washed twice, and the cell pellets were resuspended in basal medium, and then the OD_660_ was measured. The pH of cell-free culture broths was measured using a Hanna bench pH 213 meter (Woonsocket, Rhode Island, USA). The cell growth was also monitored by assessing the carbon dioxide (CO_2_) production, based on a method previously described by Darsa et al. [9]. Culture broths were titrated with 0.05 N NaOH, using 1% phenolphthalein as an indicator, and then the amount of carbon dioxide released (CO_2_ mg L^−1^) was calculated [9].

### 2.3 Scanning electron microscopy (SEM)

Cell pellets were fixed with 5% glutaraldehyde in 0.2 M potassium phosphate buffer for 24 h at room temperature and dehydrated in a graded series of ethanol for 30 min each (15%, 30%, 50%, 70%, 90%, and 100% ethanol). Gold-coated samples were examined using a JEOL JSM-6610LV scanning electron microscope (JEOL, Peabody, MA, USA) operating at 20 kV.

### 2.4 Enzyme production

Culture broths were spotted on wheat meal agar [10], starch agar [11], and carboxymethylcellulose agar [12] for protease, amylase, and cellulase production, respectively. The Petri plates were incubated for 24 h at 30°C for these qualitative analyses.

### 2.5 Sodium dodecyl sulfate-polyacrylamide gel electrophoresis (SDS-PAGE)

Extracellular and whole-cell proteins were extracted from the cell-free culture broths and cell pellets, respectively, as previously described by Stancu [13]. Extracted proteins were dissolved in Laemmli buffer and denatured at 96°C for 10 min. Protein content was determined by measuring the optical density at 280 nm (OD_280_) using a NanoDrop ND-1000 spectrophotometer (Thermo Fisher Scientific, Wilmington, DE, USA). SDS-PAGE analyses were carried out using a Minigel-Twin system (Biometra, Göttingen, Germany). After electrophoretic separation on 12% polyacrylamide gel and staining with Coomassie brilliant blue [14], the protein profiles were analyzed.

### 2.6 Polymerase chain reaction (PCR) amplification and sequencing

Genomic DNA was extracted from the cell pellets with PureLink genomic kit (Invitrogen, Carlsbad, CA, USA) and used as a DNA template for the PCR amplification of alkane hydroxylase gene of Group III encoding *alkB1* [15]. The PCR amplification of this gene was performed by using thermal Mastercycler pro S (Eppendorf, Hamburg, Germany) in a 50 µL reaction mixture containing: 0.5 µg genomic DNA, GoTaq G2 hot start polymerase (Promega, Madison, WI, USA), 5× GoTaq flexi buffer, MgCl_2_, dNTP mix, and ALK3-f/ALK3-r primers [15]. The PCR conditions included an initial denaturing step of 10 min at 94°C followed by 35 cycles of denaturing at 94°C for 1 min, annealing at 43°C for 30 s, extension at 72°C for 2 min, followed by a final extension of 10 min at 72°C. After electrophoretic separation on 1.5% TBE agarose gel [14] and staining with SYBR safe DNA gel stain (Invitrogen, Carlsbad, CA, USA), the PCR products were analyzed. The 330, 430, and 500 bp amplicons were sequenced with an ABI 3730XL at CeMIA SA (Larissa, Greece). The nucleotide sequences obtained were edited and aligned using the BioEdit software and compared with those available in the GenBank public database using the NCBI BLAST search algorithm (https://blast.ncbi.nlm.nih.gov/Blast.cgi).

### 2.7 Biosurfactant production

Emulsification activity assay was used to quantify the biosurfactant in the cell-free culture broths. Briefly, *n*-hexadecane was added to cell-free culture broths (1:2, v/v), vortexed, and allowed to stand for 24 h, and then the emulsification index (*E*_24_) was determined [16].

Biosurfactant activity assay was also used to evaluate surfactant production by this bacterium. Cell-free culture broths were dropped onto the surface of Petri plates containing water and 0.01% Sudan black in *n*-hexadecane, and then spreading of the hydrocarbon film was observed when the biosurfactant was present in the cell-free culture broths [17].

Biosurfactant was extracted from the cell-free culture broths with chloroform–methanol (2:1, v/v), as previously described by Stancu [13]. Thin-layer chromatography (TLC) analyses were carried out using a CAMAG TLC system (Muttenz, Switzerland). Biosurfactant fractions were separated on TLC precoated silica gel 60 plates (Merck) using chloroform–methanol–water mixture (65:25:4, v/v/v) as mobile phase. After derivatization with iodine vapors to detect lipids or 0.2% orcinol in 53% sulfuric acid to detect sugars in the biosurfactant molecules, TLC plates were visualized and scanned under ultraviolet (UV) light (254 nm) and visible light (500 nm).

## 3 Results

### 3.1 Growth experiments

The growth of *B. megaterium* IBB_Po17_ cells in the presence of 5% *n*-hexadecane on medium supplemented with yeast extract, proteose peptone, starch, or cellulose was monitored by measuring the optical density at 660 nm (OD_660_), pH, and CO_2_ released (Table 1). *B. megaterium* IBB_Po17_ exhibited a higher growth in the presence of *n*-hexadecane when the medium was supplemented with yeast extract, proteose peptone, or starch (OD_660_ between 1.19 and 1.26), compared with the growth on cellulose (OD_660_ 0.83). Different bacteria can grow over a wide pH range, and each bacterial strain has its own tolerance level. The optimum pH for hydrocarbon biodegradation is between 6.0 and 8.0 [9]. The pH of the growth medium for *B. megaterium* IBB_Po17_ was adjusted to 7.2. Estimating the cellular growth by measuring the pH proved a decrease in pH values ranging from 6.87 and 7.05 when the medium was supplemented with yeast extract, proteose peptone, starch, or cellulose. The production of CO_2_ by *B. megaterium* IBB_Po17_ in the presence of *n*-hexadecane was higher when the growth medium was supplemented with yeast extract, proteose peptone, or starch (CO_2_ released between 660 and 880 mg L^−1^), compared with CO_2_ released on cellulose supplemented medium (440 mg L^−1^). The acquired results showed a direct correlation between the growth of the cells, pH, and CO_2_ production. When the growth of *B. megaterium* IBB_Po17_ cells was higher, the decrease in pH values of the culture medium was higher and more amount of CO_2_ was released.

**Table 1:** Growth of *B. megaterium* IBB_Po17_ in the presence of *n*-hexadecane

Cell growth	Medium with			
	Yeast extract	Proteose peptone	Starch	Cellulose
OD_660_ (nm)	1.26	1.21	1.19	0.83
pH	6.87	6.95	6.90	7.05
CO_2_ (mg L^−1^)	660	880	660	440

Cell growth was monitored by determining the OD_660_, pH, and CO_2_ released, and the values from the table represent the average of two separate determinations.

### 3.2 SEM

**Figure 1:**
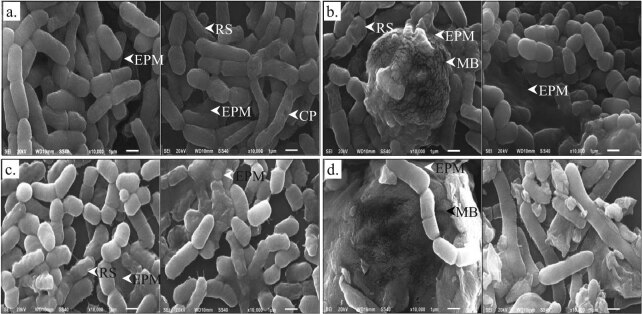
SEM studies of *B. megaterium* IBB_Po17_ grown in the presence of *n*-hexadecane. Medium with yeast extract (a), proteose peptone (b), starch (c), and cellulose (d); cell wall pores (CP), rough outer surfaces (RS), extracellular polymeric matrix (EPM), mature biofilm (MB).

SEM studies of *B. megaterium* IBB_Po17_ grown in the presence of 5% *n*-hexadecane on medium supplemented with yeast extract, proteose peptone, starch, or cellulose revealed changes in cell morphology. As observed, *B. megaterium* IBB_Po17_ grown on LB medium ranges between 1.6 and 3.8 µm in length [13]. The cell length of *B. megaterium* IBB_Po17_ grown in the presence of *n*-hexadecane on medium supplemented with yeast extract, proteose peptone, starch, or cellulose ranges between 0.8 and 5.5 µm (Figure 1a–d). A decrease in cell size was observed when *B. megaterium* IBB_Po17_ was grown in the presence of *n*-hexadecane on medium supplemented with yeast extract (approximate 0.7–1.0-fold), proteose peptone (0.6–0.7-fold), or starch (0.5–0.7-fold), as compared with the size of cells grown on LB medium. An increase in the cell size was observed in the presence of *n*-hexadecane when the medium was supplemented with cellulose (approximate 1.2–1.4-fold). Surface damages (i.e., rough outer surfaces, cell wall pores, leakage of internal contents) were observed when *B. megaterium* IBB_Po17_ cells were grown in the presence of *n*-hexadecane on medium supplemented with yeast extract, proteose peptone, or starch. No such surface damages were observed for bacterial cells grown in the presence of *n*-hexadecane on cellulose supplemented medium.

*B. megaterium* IBB_Po17_ cells grown in the presence of *n*-hexadecane on medium supplemented with yeast extract, proteose peptone, starch, or cellulose were surrounded by an extracellular polymeric matrix (EPM) (Figure 1a–d). The formation of cell clusters and mature biofilm (a three-dimensional structure) was also observed. The clustered bacterial cells were embedded in an additional EPM. The increase of EPM was observed when *B. megaterium* IBB_Po17_ was grown in the presence of *n*-hexadecane on medium supplemented with proteose peptone, starch, or cellulose.

### 3.3 Enzyme production

**Figure 2:**
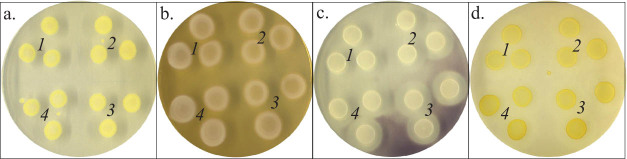
Enzyme production by *B. megaterium* IBB_Po17_ grown in the presence of *n*-hexadecane. Control (a), protease (b), amylase (c), and cellulase (d). Medium with yeast extract (1), proteose peptone (2), starch (3), and cellulose (4); confluent cell growth was observed.

*B. megaterium* IBB_Po17_ grown in the presence of 5% *n*-hexadecane on medium supplemented with yeast extract, proteose peptone, starch, or cellulose produced protease, amylase, and cellulase (Figure 2a–d). No significant differences were observed in protease, amylase, and cellulase production when the growth medium for *B. megaterium* IBB_Po17_ cells was supplemented with yeast extract, proteose peptone, starch, or cellulose.

### 3.4 SDS-PAGE

**Figure 3:**
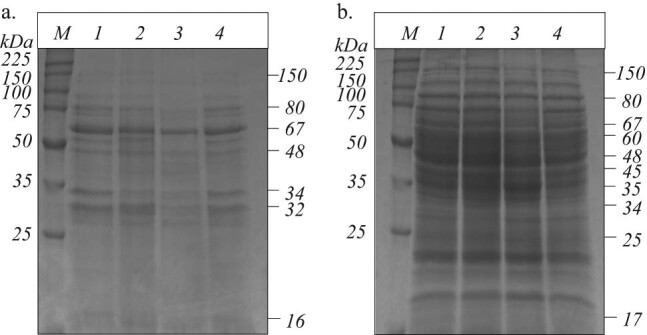
SDS-PAGE of extracellular (a) and whole-cell (b) proteins of *B. megaterium* IBB_Po17_ grown in the presence of *n*-hexadecane. Medium with yeast extract (1), proteose peptone (2), starch (3), and cellulose (4); broad range protein molecular weight marker, Promega (*M*).

The protein profiles of *B. megaterium* IBB_Po17_ cells grown in the presence of 5% *n*-hexadecane on medium supplemented with yeast extract, proteose peptone, starch, or cellulose were investigated by SDS-PAGE. *B. megaterium* IBB_Po17_ showed extracellular and whole-cell protein bands in the ranges of 16–150 kDa and 17–150 kDa, respectively (Figure 3a and b). Changes in the protein profiles of *B. megaterium* IBB_Po17_ cells were observed in the presence of *n*-hexadecane when the growth medium was supplemented with yeast extract, proteose peptone, starch, or cellulose. Extracellular proteins in the range of 32–150 kDa were produced in higher quantities by *B. megaterium* IBB_Po17_ grown in the presence of *n*-hexadecane on yeast extract, proteose peptone, or cellulose, as compared with those from cells grown on starch supplemented medium. Small alterations were also observed in the whole-cell protein profiles when *B. megaterium* IBB_Po17_ cells were grown in the presence of *n*-hexadecane on yeast extract, proteose peptone, starch, or cellulose supplemented medium.

### 3.5 PCR amplification and sequencing

**Figure 4:**
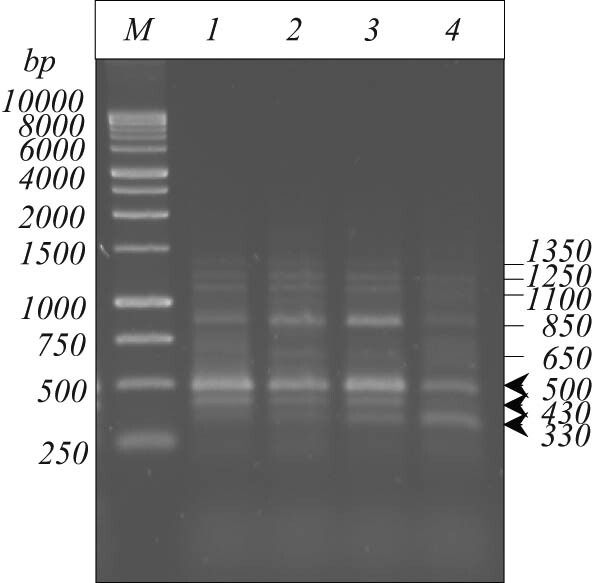
Detection of the *alkB1* gene in *B. megaterium* IBB_Po17_ grown in the presence of *n*-hexadecane. Medium with yeast extract (1), proteose peptone (2), starch (3), and cellulose (4); 1 kb DNA ladder, Promega (*M*); the positions of 330, 430, and 500 bp fragments have been marked by arrows.

Genomic DNA extracted from *B. megaterium* IBB_Po17_ cells grown in the presence of 5% *n*-hexadecane on basal medium supplemented with yeast extract, proteose peptone, starch, or cellulose was used as the template for PCR amplification of alkane hydroxylase gene of Group III encoding *alkB1*. As observed (Figure 4), the 330 bp expected amplicon for *alkB1* gene was detected in lower quantities in DNA extracted from *B. megaterium* IBB_Po17_ cells grown in the presence of *n*-hexadecane on basal medium supplemented with yeast extract or proteose peptone, and in higher quantities in DNA extracted from cells grown on starch or cellulose supplemented medium. Furthermore, seven other distinct amplicons (i.e., 430 + 500 + 650 + 850 + 1,100 + 1,250 + 1,350 bp) were obtained when the ALK3-f/ALK3-r primers for *alkB1* gene were used. The 330, 430, and 500 bp amplicons were subsequently sequenced using the amplification primers. The 330 bp fragment exhibited 88% sequence homology with alkane hydroxylase *alkB2* gene of *Pseudomonas aeruginosa* PAK (AJ633616.1). The 430 bp fragment exhibited 88% sequence homology with peptidase M20 of *B. megaterium* (WP_098332943.1), and the 500 bp fragment showed 94% sequence homology with a hypothetical protein of *B. megaterium* (WP_074898083.1).

### 3.6 Biosurfactant production

**Figure 5:**
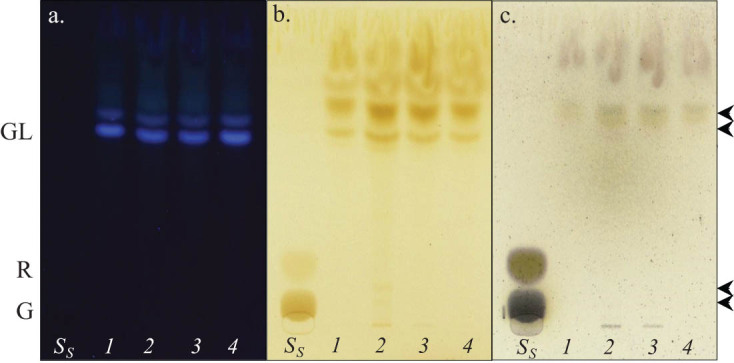
TLC of biosurfactant produced by *B. megaterium* IBB_Po17_ grown in the presence of *n*-hexadecane. TLC plate visualized under UV light (a); TLC plate stained with iodine (b) or orcinol (c) and visualized under visible white light. Medium with yeast extract (1), proteose peptone (2), starch (3), and cellulose (4); sugar standards (*S*_S_), d-glucose (G), l-rhamnose (R), glycolipids (GL); the glycolipids have been marked by arrows.

The biosurfactant production by *B. megaterium* IBB_Po17_ cells grown in the presence of 5% *n*-hexadecane on medium supplemented with yeast extract, proteose peptone, starch, or cellulose was further investigated in this study by the emulsification index (*E*_24_), biosurfactant activity, and TLC analyses. *B. megaterium* IBB_Po17_ exhibited the ability to produce biosurfactant with a good emulsification index (*E*_24_) when the growth was done in the presence of *n*-hexadecane on yeast extract (*E*_24_ 50%), proteose peptone (*E*_24_ 52%), starch (*E*_24_ 48%), or cellulose (*E*_24_ 45%) supplemented medium. Biosurfactant activity (quantified by the *n*-hexadecane spreading assay) was higher when *B. megaterium* IBB_Po17_ was grown in the presence of *n*-hexadecane on yeast extract, proteose peptone, or starch supplemented medium (diameter 83 mm), compared with the activity of biosurfactant produced by the cells on cellulose (diameter 75 mm). As observed (Figure 5a–c), two fractions were detected by TLC analyses in the cell-free culture broths of *B. megaterium* IBB_Po17_ grown in the presence of *n*-hexadecane on yeast extract (retardation factor *R*_f_ 0.50, 0.55), starch (*R*_f_ 0.49, 0.53), or cellulose (*R*_f_ 0.49, 0.54), and four fractions when the growth medium was supplemented with proteose peptone (*R*_f_ 0.08, 0.12, 0.49, 0.53). All detected fractions (*R*_f_ 0.08, 0.12, 0.49–0.50, 0.53–0.55) revealed a positive reaction with iodine (light brown spots), indicating the presence of lipid moiety, and the fourth fraction (with *R*_f_ 0.53–0.55) revealed a positive reaction also with orcinol (dark red spot), indicating the presence of sugar moiety in the biosurfactant molecules.

## 4 Discussion

*B. megaterium* strain IBB_Po17_ (GenBank KX499518) was formerly isolated from an oily sludge sample by crude oil enrichment [13]. This Gram-positive bacterium, which possesses *alkB1* and *ndoM* catabolic genes, was able to survive and grow in the presence of short-chain *n*-alkanes, such as *n*-decane, *n*-hexane, and cyclic solvents, such as cyclohexane, ethylbenzene, styrene, and toluene. As previously observed, *B. megaterium* IBB_Po17_ produced extracellular enzymes (protease, amylase, cellulase) and extracellular surfactant when the growth was done on nutrient-rich LB medium [13]. In the present study, we further investigated the ability of *B. megaterium* IBB_Po17_ to produce biosurfactant when the growth was done in the presence of 5% *n*-hexadecane on basal medium supplemented with different nitrogen (i.e., yeast extract, proteose peptone) or carbon (i.e., starch, cellulose) sources. The long-chain *n*-alkane, *n*-hexadecane, was used as the hydrophobic carbon source in order to evaluate the degradative potential of *B. megaterium* IBB_Po17_.

*B. megaterium* IBB_Po17_ revealed a higher growth in the presence of *n*-hexadecane when the medium was supplemented with yeast extract, proteose peptone, or starch, compared with the growth on cellulose. Nitrogen and carbon are the most important media components that act as essential stimulants for the growth of bacteria and also for the enzyme production [18]. Estimating the cellular growth by measuring the pH proved a decrease in pH values when the medium was supplemented with yeast extract, proteose peptone, starch, or cellulose, indicating the formation of organic acids (Table 1). Similar results were obtained by Darsa et al. [9] for another Gram-positive strain, *B. subtilis*, which was isolated from a soil sample collected from petrol bunks and workshops. The petrol utilization by *B. subtilis* induced changes in the optical density and pH values of the growth medium due to the production of organic acids. These acidic metabolic products are responsible for the decrease in pH of the growth medium [9].

The mineralization studies, which involve the measurements of total CO_2_ released, provide excellent information which confirms the active hydrocarbon degradation in polluted soils [19]. The production of CO_2_ by *B. megaterium* IBB_Po17_ in the presence of *n*-hexadecane was higher when the growth medium was supplemented with yeast extract, proteose peptone, or starch, compared with CO_2_ released on cellulose supplemented medium. Similarly, Darsa et al. [9] proved changes in the levels of CO_2_ released in the growth medium during petrol degradation by a *B. subtilis* strain.

Changes in cell morphology (e.g., cell sizes, cell surfaces) were observed when *B. megaterium* IBB_Po17_ was grown in the presence of *n*-hexadecane on medium supplemented with yeast extract, proteose peptone, starch, or cellulose (Figure 1a–d). There are several reports in which the cell sizes are decreasing or increasing when bacteria are exposed to different stressful conditions [20,21,22]. The surface–volume ratio of the cells is the most important factor responsible for cell size changes. The reduction in the cell surface represents an effective mechanism of the cells to reduce the toxic effect of environmental stress factors by reducing the attachable surface in relation to the whole-cell volume [20]. Therefore, it was not surprising to observe cell size changes in *B. megaterium* IBB_Po17_ grown in the presence of *n*-hexadecane on yeast extract, proteose peptone, starch, or cellulose supplemented medium. Cell morphology alterations, which implicate modification in the cell membrane structure, alteration of the cell surface properties (e.g., permeability barrier, energy transducer), and metabolism, were reported in Gram-positive bacteria, including in different *Bacillus* strains, as a response to different environmental stresses (e.g., organic solvents). An increase in cell membrane permeability is considered the main reason for cell death [22].

Increase of EPM involved in biofilm formation was observed when *B. megaterium* IBB_Po17_ was grown in the presence of *n*-hexadecane on medium supplemented with yeast extract, proteose peptone, starch, or cellulose (Figure 1a–d). Several *Bacillus* strains were able to form biofilms on different surfaces, and these bacteria are more resistant to environmental stress [23]. During biofilm development, cell morphological changes occur. Initially, the cells are short and motile, while in mature biofilm, the cells form long chains of nonmotile rods which adhere to each other and on the surfaces by secreting an EPM (mainly polysaccharides, proteins, and nucleic acids) [23].

No significant differences were observed in protease, amylase, and cellulase production when the growth medium for *B. megaterium* IBB_Po17_ cells was supplemented with yeast extract, proteose peptone, starch, or cellulose (Figure 2a–d). The production of extracellular enzymes is generally influenced by media components (e.g., nitrogen and carbon source) and several other factors (e.g., temperature, pH, aeration, incubation time). There is no defined medium for the best production of extracellular enzymes from different *Bacillus* strains [24,25,26]. Each strain has its unique conditions for maximum production of the extracellular enzymes, such as protease [18,24,27], amylase [28], and cellulase [25].

Changes in the protein profiles (including the 48, 67, and 80 kDa bands) of *B. megaterium* IBB_Po17_ cells were observed in the presence of *n*-hexadecane when the growth medium was supplemented with yeast extract, proteose peptone, starch, or cellulose (Figure 3a and b). Ahmetoglu et al. [26], Tiwari et al. [28], and Gaur and Tiwari [25] reported that the molecular weights of the proteases, amylase, and cellulase from different *Bacillus* strains (i.e., *Bacillus* sp. KG5, *Bacillus tequilensis* RG-01, *Bacillus vallismortis* RG-07) were 48, 67, and 80 kDa, respectively.

In this study, the *alkB1* gene (330 bp amplicon) was detected in lower quantities in DNA extracted from *B. megaterium* IBB_Po17_ cells grown in the presence of *n*-hexadecane on basal medium supplemented with yeast extract or proteose peptone, and in higher quantities in DNA extracted from cells grown on starch or cellulose supplemented medium (Figure 4). Seven other distinct amplicons were also obtained by PCR when the primers for *alkB1* gene were used.

*B. megaterium* IBB_Po17_ exhibited the ability to produce biosurfactant with a good emulsification index when the growth was done in the presence of *n*-hexadecane on yeast extract, proteose peptone, starch, or cellulose supplemented medium. A direct correlation between the growth of the cells and biosurfactant production was observed. Biosurfactant activity was higher when *B. megaterium* IBB_Po17_ was grown in the presence of *n*-hexadecane on yeast extract, proteose peptone, or starch supplemented medium, compared with the activity of biosurfactant produced by the cells on cellulose. Two fractions were detected by TLC analyses in the cell-free culture broths of *B. megaterium* IBB_Po17_ grown in the presence of *n*-hexadecane on yeast extract, starch, or cellulose, and four fractions when the growth medium was supplemented with proteose peptone. All detected fractions showed a positive reaction with iodine, indicating the presence of lipid moiety, and one of the fractions showed a positive reaction also with orcinol, indicating the presence of sugar moiety in the biosurfactant molecules (Figure 5a–c). Based on the chemical composition, most of the biosurfactants produced by different *Bacillus* species were lipopeptides [5,7,16,29,30]. However, Thavasi et al. [31] reported that the biosurfactant produced by a *B. megaterium* strain was classified as a glycolipid with 28% carbohydrate and 70% lipid in its structure.

## 5 Conclusions

*B. megaterium* IBB_Po17_ revealed a higher growth in the presence of *n*-hexadecane when the medium was supplemented with yeast extract, proteose peptone, or starch, compared with the growth on cellulose. When the growth of *B. megaterium* IBB_Po17_ cells was higher, the decrease in pH values of the medium was higher, too, and more amount of CO_2_ was released. Changes in cell morphology and protein profiles were observed when *B. megaterium* IBB_Po17_ was grown in the presence of *n*-hexadecane on medium supplemented with yeast extract, proteose peptone, starch, or cellulose. A direct correlation between the growth of the cells and biosurfactant production was observed. Based on our data collected so far, *B. megaterium* IBB_Po17_ could be a promising candidate for the bioremediation of petroleum hydrocarbon polluted environments.
